# Purposeful microbiology comment added to urine cultures with *Staphylococcus aureus* increases orders for follow-up blood cultures

**DOI:** 10.1099/acmi.0.000224

**Published:** 2021-04-15

**Authors:** Donald Brody Duncan, Yasmeen Marbaniang Vincent, Cheryl Main

**Affiliations:** ^1^​ Department of Pathology and Molecular Medicine, McMaster University, Hamilton, Ontario, Canada; ^2^​ Hamilton Regional Laboratory Medicine Program, Hamilton Health Sciences, Hamilton, Ontario, Canada

**Keywords:** bacteriuria, blood culture, comment, *Staphylococcus aureus*, urine

## Abstract

**Introduction:**

Patients with *Staphylococcus aureus* bacteriuria (SABU) often have underlying invasive disease, including *S. aureus* bacteremia (SAB). It has been proposed that most patients with SABU should have a blood culture done to rule out SAB. A preliminary audit suggested that our local hospitals had a low rate of follow-up blood culture orders for patients with SABU. In response to this, our microbiology laboratory changed the comment appended to urine cultures with growth of *S. aureus* to make a more assertive link between SABU and SAB and to recommend follow-up blood cultures.

**Aim:**

We designed a retrospective quasi-experimental study to see if the change in microbiology comment wording had an effect on clinician behaviour. We hypothesized that this simple comment change to make a more assertive link between SABU and SAB would lead to an increase in follow-up blood culture orders.

**Methodology:**

We used microbiology records to identify adult patients with urine cultures positive for *Staphylococcus aureus* at three acute-care hospitals in Hamilton, Ontario, Canada, for 1 year pre- and post-intervention. We recorded urine and blood culture results, timing, patient demographics, and in-hospital mortality.

**Results:**

A total of 243 adult patients with urine cultures with *S. aureus* were identified for inclusion. The primary outcome was met, as there was a significant increase in blood culture orders between the pre-intervention and post-intervention groups (66.9 % vs 80.4 %). This difference was mainly driven by an increase for emergency department and urgent care patients (30.6 % vs 63.6 %). The inpatient group had a high baseline rate of blood culture orders that did not change significantly (80.0 % vs 84.7 %). There was no significant change in detection of SAB (23.5 % vs 32.7 %) or inpatient mortality (18.0 % vs 24.7 %).

**Conclusion:**

Our study shows that a simple, purposeful comment appended to urine cultures with *S. aureus* leads to a significant increase in follow-up blood culture orders.

## Introduction

*Staphylococcus aureus* bacteriuria (SABU) is an uncommon clinical finding [1]. Although it often represents asymptomatic colonization, a significant minority of patients with SABU have invasive disease, including *S. aureus* bacteremia (SAB) [2]. Rates of SAB in patients with SABU range from 6.8–21 %, depending on the setting and patient population [3–5]. This includes patients with primary urinary tract infection and those with non-urinary invasive disease with secondary bacteriuria [6–8]. Previously identified risk factors for SAB in patients with SABU include inpatient status, male sex, methicillin-resistant *S. aureus* (MRSA), prior urinary procedures, gross hematuria, signs of sepsis, and lack of urinary symptoms [5, 9, 10]. However, none of these are sufficient to rule in or out invasive *S. aureus* disease. For this reason, it has been suggested that most patients with SABU should have a blood culture done to rule out SAB [3, 9].

At our own institution, a preliminary audit of microbiology records suggested that our local hospitals and their associated emergency departments and urgent care centres (ED/UC) had a low rate of follow-up blood culture orders for patients with SABU. Prior to the audit, all urine cultures positive for *S. aureus* had the following comment appended to the report: ‘Primary *Staphylococcus aureus* urinary tract infections are uncommon. Please submit blood cultures if clinically indicated.’ As part of a quality improvement (QI) initiative, this comment was subsequently changed to be more directed and purposeful: ‘* Staphylococcus aureus* bacteriuria is associated with *S. aureus* bacteremia. Blood cultures are recommended.’

Purposeful, directed comments added to microbiology reports can lead to changes in antibiotic prescribing behaviour of clinicians [11, 12]. Adding a comment specifically to *S. aureus* urine cultures has been suggested, but has not been studied for efficacy [5]. As such, we designed a retrospective quasi-experimental study to see if the change in microbiology comment wording had an effect on clinician behaviour. We hypothesized that this simple comment change to make a more assertive link between SABU and SAB would lead to an increase in follow-up blood culture orders.

## Methods

### Study design and intervention

This quasi-experimental before-and-after study included patients with urine cultures positive for *Staphylococcus aureus* at three acute-care hospitals in Hamilton, Ontario, Canada and their associated emergency departments and urgent care centres. The study was divided into two 12 month periods: pre-intervention (1 August 2018–31 July 2019) and post-intervention (1 August 2019–31 July 2020). The comment change described above was applied to all urine culture reports with growth of *S. aureus* starting on 1 August 2019. A laboratory memo was sent to all clinical areas to inform them of the change and its rationale. No additional education was provided.

### Study population

Microbiology records were used to identify patients with urine cultures with growth of *S. aureus* from 1 August 2018 to 31 July 2020. The study population included adults, at least 18 years of age, who had urine cultures taken in the emergency department, urgent care centre, or inpatient ward. Only the first positive urine culture during the study period was included for analysis.

Exclusion criteria included urine cultures taken on non-acute medical care units (palliative care, rehabilitation, mental health) and during urologic day-procedures without subsequent hospital admission. Infection control surveillance cultures for MRSA were also excluded.

### Microbiology

All microbiological work was done at the Hamilton Regional Laboratory Medicine Program. Urine cultures were plated on CHROMagar Orientation media (CHROMagar, Paris, France) and growth was considered significant if ≥10^7^ c.f.u. l^-1^. Urine cultures with growth <10^7^ c.f.u. l^-1^ were reported as ‘no significant growth’ without further work-up, and could not be identified for inclusion in this study. Cultures reported as multiple organisms including *S. aureus* were included. Urine culture results for both inpatients and discharged patients were communicated to the most responsible physician for that ward or ED/UC.

### Study data

Basic demographic information including age, sex, and unit of admission were recorded. The date of the first urine culture positive for *S. aureus* was used to group patient to the pre-intervention or post-intervention groups. Blood culture orders within 30 days of the positive urine culture were recorded.

Comparisons were made between the pre-intervention and post-intervention groups. Data was analysed for the entire cohort and for inpatient and ED/UC subgroups. Patients who had urine cultures taken in the emergency department but were admitted during that same visit were included in the inpatient subgroup.

The primary outcome was the proportion of patients with SABU who had blood cultures ordered within 30 days. Secondary outcomes included detection rate of SAB in patients with SABU; in hospital mortality during that admission for inpatients; prevalence of MRSA; and proportion of patients who had blood cultures done ‘early’, defined as within 2 days of urine culture for inpatients and 5 days for ED/UC patients. Different time frames were chosen due to inherent differences in clinician follow-up and patient availability between inpatient and ED/UC settings.

### Statistical analysis

Statistical analysis was done using R version 3.6.2 [13]. All data for the primary and secondary outcomes were categorical and compared using Chi-square test or Fisher’s exact test as appropriate. Differences in average age between groups were compared using the *T*-test.

### Ethics

This project was reviewed and approved by the Hamilton Integrated Research Ethics Board. Individual patient consent was not required. The intervention was planned and implemented as a stand-alone quality improvement project, independent from the planning of this retrospective study.

## Results

Review of our microbiology database identified 346 non-surveillance urine cultures positive for *S. aureus* from adults patients in acute-care hospitals and their ED/UC between 1 August 2018 and 31 July 2020. We excluded 36 specimens as they were collected from non-acute medical care units (rehabilitation, mental health) or urologic day procedures. That left 310 urine culture specimens remaining, which corresponded to 243 unique patients. Based on the date of their first urine culture, they were divided into 136 patients in the pre-intervention group and 107 patients in the post-intervention group. There were no significant differences in sex, age distribution, or rate of MRSA or mixed urine culture growth between the two groups (Table 1). One patient’s urine isolate was not tested for antibiotic susceptibilities, but they had *S. aureus* isolates from other body sites that were methicillin-susceptible, so the urine isolate was assumed to not be MRSA.

**Table 1.** Baseline characteristics of patients with SABU, pre- and post-intervention

	Pre-intervention (*n*=136)	Post-intervention (*n*=107)	*P* value
Average age in years (standard deviation)	67.7 (19.1)	68.7 (17.8)	0.668
Male sex	88 (64.7 %)	63 (58.9 %)	0.352
ED/UC vs inpatient	36 (26.5 %) vs 100 (73.5 %)	22 (20.6 %) vs 85 (79.4 %)	0.283
Mixed urine culture	42 (30.9 %)	33 (30.8 %)	0.630
MRSA	49 (36.0 %)	29 (27.1 %)	0.139

Outcome measures for the pre-intervention and post-intervention groups were then compared (Table 2). For the primary outcome, there was a significant increase in blood culture orders in patients with SABU after the comment change (66.9 % vs 80.4 %, *P*=0.019). This increase was almost entirely in patients who attended the ED/UC (30.6 % vs 63.6 %, *P*=0.014). There was no significant difference in blood culture orders for patients with SABU on the inpatient wards (80.0 % vs 84.7 %, *P*=0.405). A large proportion of patients had blood cultures drawn on the same day or before the positive urine culture (36.0 % of patients pre-intervention, 42.9 % of patients post-intervention), therefore those patients were excluded and the data reanalysed. The same result was found, with a significant increase overall in blood culture orders after SABU (48.3 % vs 65.6 %, *P*=0.037) which was attributed to the ED/UC (30.6 % vs 57.9 %, *P*=0.049) with no significant difference on the inpatient wards (60.8 % vs 69.0 %, *P*=0.407).

**Table 2.** Outcomes for patients with SABU, pre- and post-intervention

	Pre-intervention (*n*=136)	Post-intervention (*n*=107)	*P* value
Blood culture done			
Overall	91 (66.9 %)	86 (80.4 %)	0.019
ED/UC	11/36 (30.6 %)	14/22 (63.6 %)	0.014
Inpatient	80/100 (80.0 %)	72/85 (84.7 %)	0.405
Blood culture done (excluding if blood culture done at same time or before urine culture)			
Overall	42/87 (48.3 %)	40/61 (65.6 %)	0.037
ED/UC	11/36 (30.6 %)	11/19 (57.9 %)	0.049
Inpatient	31/51 (60.8 %)	29/42 (69.0 %)	0.407
Blood culture done ‘early’ (≤2 days for inpatient, ≤5 days for ED/UC)			
Overall	81 (59.6 %)	74 (69.2 %)	0.122
ED/UC	8/36 (22.2 %)	12/22 (54.5 %)	0.012
Inpatient	73/100 (73.0 %)	62/85 (72.9 %)	0.993
SAB detected			
Overall	32 (23.5 %)	35 (32.7 %)	0.112
ED/UC	1/36 (2.8 %)	2/22 (9.0 %)	0.551
Inpatient	31/100 (31.0 %)	33/85 (38.8 %)	0.265
Death			
Inpatient	18/100 (18.0 %)	21/85 (24.7 %)	0.265

There was no significant difference in secondary outcomes between the pre-intervention and post-intervention groups, including no difference in detection of SAB (23.5 % vs 32.7 %, *P*=0.112) or inpatient mortality during that admission (18.0 % vs 24.7 %, *P*=0.265). For the ED/UC subgroup only, there was an increase in the proportion of blood cultures taken ‘early’ within 5 days of the urine culture with *S. aureus* (22.2 % vs 54.5 %, *P*=0.012).

To better understand the differences between the ED/UC and inpatient subgroups, their baseline characteristics and outcomes were compared (Table 3). Inpatients with SABU were on average older (70.1 years vs 62.1 years, *P*=0.015), had a higher rate of blood cultures done (82.2 % vs 43.1 %, *P* <0.001), and had a higher rate of SAB detected (34.6 % vs 5.2 %, *P* <0.001) compared with ED/UC patients. There was no significant difference in sex distribution, or rate of MRSA or mixed urine culture growth.

**Table 3.** Baseline characteristics and outcomes for ED/UC and inpatient subgroups

	ED/UC (*n*=58)	Inpatient (*n*=185)	*P* value
Age in years (standard deviation)	62.1 (22.5)	70.1 (16.6)	0.015
Male sex	35 (60.3 %)	116 (62.7 %)	0.747
Mixed	16 (27.6 %)	56 (30.3 %)	0.696
MRSA	15 (25.9 %)	63 (34.1 %)	0.244
Blood culture done	25 (43.1 %)	152/(82.2 %)	<0.001
Blood culture done ‘early’ (≤2 days for inpatient, ≤5 days for ED/UC)	20 (34.5 %)	135 (73.0 %)	<0.001
SAB detected	3 (5.2 %)	64 (34.6 %)	<0.001

Overall crude in-hospital mortality was 21.1 % for inpatients with SABU. As a post-hoc analysis, we examined mortality rates among the entire inpatient cohort to see if there were significant differences based on microbiologic investigations and results. Mortality was numerically lower for inpatients who did not have blood cultures ordered compared to those who did, but this finding was not statistically significant (9.1 % vs 23.7 %, *P*=0.096). Patients with blood cultures positive for *S. aureus* had significantly higher mortality than patients with SABU without documented SAB (29.7 % vs 16.5 %, *P*=0.037). There was no significant difference in mortality between patients with MRSA vs methicillin-susceptible *S. aureus* bacteriuria (20.5 % vs 22.2 %, *P*=0.785) or with pure vs mixed urine cultures (19.4 % vs 25.0 %, *P*=0.389).

## Discussion

Our study shows that a purposeful, directed comment appended to urine cultures positive for *S. aureus* increased the rate of blood culture orders for patients with SABU. One strength of our study is that this increase was significant despite the fact that our laboratory already had a less-assertive comment appended to these urine culture reports; we suspect that laboratories that initially do not have any comment on urine cultures positive for *S. aureus* would find an even greater increase after implementation of this change. Another strength of this study is that we did not supply additional education to clinicians about SABU other than a memo when the change was made. This validates the finding that simple, low-cost changes in microbiology reporting can have significant impact on clinician behaviour [11, 14–16].

This difference in blood culture orders occurred mainly in the ED/UC population, and there was no significant change in blood culture orders for inpatients after the intervention. There was already a fairly high rate of blood culture orders for inpatients with SABU before the intervention, which can in part be attributed to the prior comment appended to *S. aureus* urine cultures. It is possible that alternative methods of communication would be more appropriate for clinicians working in inpatient areas where the majority of patients with SABU already receive follow-up blood cultures. Because our study was limited to microbiologic data, we did not look at other patient factors that may influence the decision to order blood cultures, including presence of signs and symptoms of infection, comorbidities, and the patient’s goals of care.

However, for inpatients with SABU that did not have blood cultures ordered within 30 days, mortality during that admission was 9.1 %. Although this is numerically smaller than the 23.7 % mortality for patients who did have blood cultures ordered, it is still a notably high mortality rate. This does not prove a causal relationship between SABU and death, but without blood cultures it is impossible to tell if any of that inpatient mortality could be attributed to undetected SAB. It is well established in the literature that SABU should not be assumed to be a benign finding, as it is associated with complicated invasive *S. aureus* infections and increased mortality [4, 17, 18]. We suggest that blood cultures be considered for all inpatients with SABU, including patients lacking overt infectious symptoms.

There was no significant change in detection of SAB after the intervention. This is likely related to two factors: the low event rate of SAB for ED/UC patients, and the unchanged rate of blood culture orders for inpatients. Our study is underpowered to determine if the significant increase in blood culture orders for ED/UC patients would lead to an increase in detection of SAB, as there were only three cases of SAB in ED/UC patients during the 24 month study period. The retrospective, hospital-based nature of our study also limited the information we could gather on ED/UC patients. It is possible they had further follow-up in other outpatient settings, or required admission or died at a healthcare facility outside of our study area. Because of the overall low rate of SAB among ED/UC patients with SABU in our study, it is not clear if these patients should routinely have follow-up blood cultures done. The only study on SABU at the population level showed that outpatients with SABU, including emergency department patients, were less likely to have SAB compared to inpatients [5]. The prevalence of SAB in outpatients with SABU in that study (4.3 %) was similar to our findings in ED/UC patients (5.2 %). Possible harms of routine blood cultures for ED/UC patients with SABU include the need for the patient to return to the healthcare setting after discharge, increased costs, iatrogenic anaemia, and the risk of unnecessary antibiotic treatment for the small number of patients with positive blood cultures due to contamination rather than true invasive disease [19]. Nonetheless, we believe that doing blood cultures in these patients is justified given the morbidity and mortality associated with SABU+SAB. Further study is needed on the appropriate evaluation of ED/UC patients with SABU, including if the presence of risk factors or symptoms impacts the utility of blood cultures [3].

An important limitation is that our microbiology laboratory is hospital-based and does not process specimens from the community. We also excluded patients from non-acute medical wards, including palliative care, rehabilitation, and mental health, as well as children less than 18 years old. As such, these results cannot be assumed to apply to those patient populations. There is no clear evidence in the literature supporting routine investigation for SAB in outpatients with SABU, especially those without risk factors or symptoms of sepsis.

Our laboratory does not routinely identify urine culture isolates with growth <10^7^ c.f.u. l^-1^. Although this cut-off decreases the time and resources spent on identifying non-pathogenic flora, it also means a small number of true pathogens will be missed. A wide variety of colony count cut-offs for reporting SABU are described in the literature [3], and there is no evidence that the amount of growth in urine culture is associated with the incidence of invasive disease [4, 5]. We cannot rule out that the inability to identify patients with low-level bacteriuria could have affected our study results.

Another limitation is the non-controlled, non-randomized nature of our quasi-experimental study. It is difficult to prove causality due to the possibility of unmeasured confounders or regression to the mean. It would be challenging to design a controlled, randomized trial to investigate this question, given that microbiology reporting is typically uniform for all hospitals served by a single laboratory. Further research from other laboratories implementing similar changes will be needed to establish a stronger evidence base for the benefit of adding simple microbiologic comments to urine cultures positive for *S. aureus *.

## Conclusion

Appending a purposeful, directed comment to urine cultures positive for *S. aureus* was associated with an increased rate of blood culture orders for patients with SABU. This increase was mostly among outpatients with SABU in emergency departments and urgent care centres, as inpatients with SABU already had a high rate of follow-up blood cultures at our local institutions. Given the high mortality for inpatients with SABU, we propose that all microbiology laboratories should consider routinely adding a simple comment suggesting follow-up blood cultures to all urine cultures positive for *S. aureus *.
